# Reference values for the Teller Acuity Cards II (TAC II) in infants and preverbal children, a meta‐analysis

**DOI:** 10.1111/aos.17447

**Published:** 2025-01-28

**Authors:** Catelijne M. Neijzen, Femke M. de Wit, Ymkje M. Hettinga, Joke H. de Boer, Maria M. van Genderen, Gerard C. de Wit

**Affiliations:** ^1^ Bartiméus Diagnostic Center for Complex Visual Disorders Zeist The Netherlands; ^2^ Department of Ophthalmology University Medical Center Utrecht Utrecht The Netherlands; ^3^ Radboud University Nijmegen The Netherlands

**Keywords:** Infants and preverbal children, Preferential looking, Reference values, TAC II, Teller Acuity Cards, Visual acuity

## Abstract

**Purpose:**

The Teller Acuity Card (TAC) procedure is a preferential‐looking method to assess visual acuity in infants and preverbal children and provides a quantitative measure of grating acuity. Several studies containing reference values have been published, the majority based on an older version of the TAC card set. In 2003, a new version of the TAC was released, called the TAC II. This study aims to provide clinicians with a unified set of age norms for the TAC II.

**Methods:**

We conducted a literature search and extracted individual data points per age from either tables or figures. We performed a weighted regression on both the standard deviation and the mean visual acuity as a function of age. Furthermore, based on literature, we corrected data points from studies using the original TAC by subtracting 0.3 octave to align them with the TAC II age norms.

**Results:**

A total of 5 studies, published between 1990 and 2006, provided binocular visual acuity data for 837 children and monocular visual acuity data for 1052 children. Age norms were derived from these combined data sets.

**Conclusions:**

The TAC charts are the gold standard for assessing visual acuity between the ages of 0 and 36 months. We provide a unified set of age norms for TAC II, the most recent version of the TAC charts.

## 1 INTRODUCTION

The development of normal vision is a complex process that depends on the maturation of both the eye and the brain. Infant vision develops rapidly in the first months of life, followed by a more gradual improvement (Dirch et al., 1988; Rios Salomao and Fix Ventura, 1995). Measuring visual acuity in very young children is challenging as they are unable to provide verbal responses. In the year 1958, Fantz and colleagues demonstrated that visual acuities at a young age could be quantified by observing that children have a natural preference for looking at patterned stimuli rather than a blank, homogeneous space (Fantz, 1958; Teller et al., 2003). In 1985, Teller and McDonald expanded upon this principle of preferential looking and devised the acuity card procedure, which was initially employed in a laboratory setting (McDonald et al., 1986). Subsequently, Teller began commercially producing the Vistech‐Teller Acuity Cards (TAC or TAC I) as a rapid measurement technique for clinical use. The TAC I comprised stripes of varying spatial frequency, as were also used in earlier prototype cards. In the original test setup, the child was seated on the parent's lap, with the TAC card placed in a designated stage (i.e., a grey card box designed to minimise environmental distractions). In clinical practice, however, the TAC set is primarily used without this stage. The examiner uses the cards to subjectively evaluate the child's responses to the stimuli, including eye movements, fixation, and head movements (Teller et al., 2003).

The term ‘grating acuity’ refers to a specific measure of visual acuity, defined by the finest stripe pattern (highest spatial frequency) that the infant can resolve centrally or slightly peripherally. This detection visual acuity is not exactly the same as the recognition visual acuity as measured with optotypes in older children and adults (Kushner et al., 1995). The TAC procedure, proving a grating acuity, is easy to perform as it requires no complicated equipment, testing is quite rapid and non‐invasive, and clinical success rates are high (Mayer et al., 1995; Quinn et al., 1993; Teller et al., 1986). It is widely accepted as the standard vision assessment for infants and preverbal children. Vistech Consultants, Inc. manufactured the original Teller Acuity Cards, but in 2003 they ceased production. Subsequently, the original developers worked with Stereo Optical Co. to produce a newer version: TAC II (Teller et al., 2003). Reference values provided by the manufacturer in the manual include separate binocular TAC I norms based on Rios Salomao and Fix Ventura (1995) and Courage and Adams (1990) and separate monocular norms based on Mayer et al. (1995), Rios Salomao and Fix Ventura et al. (1995), Courage and Adams (1990), Mayer et al. (1995) and Rios Salomao and Fix Ventura (1995). The integration of data from these studies into a single, practical reference set for utilisation in clinical settings for TAC II has not yet been realised. Also, since then, new studies on reference values have been conducted. This study aims to provide clinicians with a single set of TAC II reference values in children based on the available literature, both binocularly and monocularly.

## 2 METHODS

### 2.1 Datacollection

A search was conducted in PubMed to identify papers providing age norms for the TAC I and TAC II for infants and preverbal children. We used a combination of keywords and MeSH terms related to the topic. Additionally, the reference lists of relevant reviews and articles were manually searched to identify any further studies that might have been missed during the initial search. Inclusion criteria were as follows: (1) the children tested were in overall good health, that is, had no neurological, developmental, or structural ophthalmic abnormalities, (2) the mean visual acuity (VA) and standard deviations (SD) were provided in the logarithmic domain and not in the linear domain, as previous research showed that visual acuity is approximately normally distributed in the logarithmic domain (Lam et al., 1996; McDonald et al., 1986; Ohlsson & Villarreal, 2005) and (3) individual data points were available preferably in a table, if not, the program ‘PlotDigitizer Online’ was used to extract data from suitable figures. A study was excluded if it did not use a Teller acuity stage. The following data were extracted: article characteristics (author, publishing dates), study characteristics: at different ages, the sample sizes (*n*), usage of TAC I and/or TAC II, mean visual acuities and standard deviations/standard error of the mean (SEM)/tolerance limits in log scale.

### 2.2 Analysis

If a standard error of the mean (SEM) was given instead of a standard deviation, the following formula was used to obtain the SD:

(1) SD=SEM×√n

with *n* the number of subjects. If tolerance limits were given, the standard deviation was obtained according to:

(2) $$\mathrm{SD}=\frac{{\mathrm{TL}}_{\text{upper}}-{\mathrm{TL}}_{\text{lower}}}{2k}$$

with TL_upper_ the upper tolerance limit, TL_lower_ the lower tolerance limit and k the k‐factor using the function K in R with the Howe (HE) method. Given the lack of sufficient data on TAC II, the decision was made to utilise both sets of data collected using TAC I and TAC II.

In our analysis, we corrected for the different sizes of study groups in the different studies by performing a weighted regression on both the standard deviation of the log visual acuity and the log mean visual acuity as a function of log age. For the standard deviation, we used a linear regression model. For the mean visual acuity, we used a restricted cubic splines model with 3 knots evenly distributed over the age range of 3–36 months in the log domain (5.6, 10.4 and 19.3 months). We did not distribute the knots evenly over the full age range because that would have resulted in overfitting at the lower ages due to the limited amount of data in the range of 0.5–3 months. To verify the model fit and the assumption of normally distributed residuals, plots of the residuals and Q–Q plots were inspected visually.

Literature shows that the TAC I gives a higher visual acuity outcome than the TAC II. Clifford et al. (2005) observed a significant difference of 0.2 octave at 3.5 months, 0.4 octave at 11 months, and 0.7 octave at 30 months between the TAC I and TAC II cards (Clifford et al., 2005). The authors discuss that the 0.7 octave at 30 months is probably too high because of an edge artefact in one of the TAC I cards. If we take the average of the remaining two measurement points, the TAC I gives 0.3 octave higher values than the TAC II. A similar value was obtained by Kasugai et al. (2006), who found a significant difference of 0.41 octaves in children who scored better than 6.5 cy/cm, while a lesser difference of 0.25 octaves was observed in children who scored below that threshold (Kasugai et al., 2006). Since our goal is to determine unified reference limits for the TAC II, all datapoints based on the TAC I were reduced by 0.3 octave.

The 95% upper and lower prediction interval limits of the log visual acuity were determined according to:

(3) $$\text{Lower limit}\;\left(\mathrm{age}\right)=\text{mean}\;\mathrm{VA}\;\left(\mathrm{age}\right)–1.96\cdot \mathrm{SD}\;\left(\mathrm{age}\right)$$

(4) $$\text{Upper limit}\;\left(\mathrm{age}\right)=\text{mean}\;\mathrm{VA}\;\left(\mathrm{age}\right)+1.96\cdot \mathrm{SD}\;\left(\mathrm{age}\right)$$

with the mean VA and SD (both in the log domain) as a function of age resulting from the regression analyses. To assess if the SD changes significantly with age, a *t*‐test was performed on the fitted slope. A *p*‐value of 0.05 or less was considered to be statistically significant. All analyses were performed using R (version 4.3.1) and R Studio (version 2024.04.2) with the readxl and splines libraries.

## 3 RESULTS

Table 1 shows the summary of full‐text reviewed papers. Following the application of inclusion and exclusion criteria, a total of 5 studies were included in this meta‐analysis. Papers were published between 1990 and 2006 with sample sizes ranging from 8 to 44 participants and a total number of 837 included binocular TAC assessments and 1052 monocular TAC assessments. Xiang et al. (2021) did meet the pre‐established inclusion criteria; however, we excluded this study from analysis because the results were significantly divergent from those of other papers. Because Courage and Adams (1990) did not use a Teller acuity stage below 6 months of age, their data could only be included from this age forward. The data point at 30 months of Clifford‐Donaldson et al. (2006) was also excluded from analysis since the author questioned the validity of this point due to the presence of an edge artefact on a specific TAC I card. The edge artefact was not present in the TAC II cards, so the data point at 30 months from Clifford et al. (2005) was not excluded. There were no data points <1 month for analysing the monocular data. Visualisation of results of all full‐text reviewed studies is shown in Figure S1.

**TABLE 1:** Summary of full‐text reviewed papers.

Paper	Country of origin	Eyes	Methods		Comments	Included
			TAC	Stage		
Courage and Adams (1990)	Canada	140 (ODS), 80 (ODS) included	TAC I	Used in children older than or equal to 6 months	Data with and without stage (<6 months excluded)	Yes, partially
Mayer et al. (1995)	USA	428 (OD/OS)	TAC I	Yes	The author did not use the 38 cy/cm TAC card because of edge artefact	Yes
Rios Salomao and Fix Ventura (1995)	Brazil	646 (ODS), 637 (ODS) included, 624 (OD/OS)	TAC I	Yes	ODS data point at 2 months excluded because outlier	Yes
Grunewald and Mayer (1997)	Germany	98 (ODS), 41 (OD/OS)	TAC I	No	Assumed same SD for all ages, no stage	No
Cavallini et al. (2002)	Italy	60 ODS	TAC I	No	Standard deviations were not determined in the log domain, subjects were followed over time, no stage	No
Clifford et al. (2005)	USA	60 (ODS)	TAC I and II	Yes	Only TAC II data included	Yes
Clifford‐Donaldson et al. (2006)	USA	80 (ODS), 60 included (ODS)	Presumably TAC I	Yes and no	Data point at 30 months of age was excluded; only data with usage of stage included	Yes
Leone et al. (2014)	Australia	509 (ODS), 411 (OD/OS)	TAC II	No	No stage was used	No
Xiang et al. (2021)	China	218 (ODS), 208 (OD/OS)	TAC II	Yes	Tolerance limits used; data too divergent from other studies	No
Sarbajna et al. (2024)	India	75 (ODS), 75 (OS/OD)	TAC II	Yes	Standard deviations not determined in log domain	No

**FIGURE 1:**
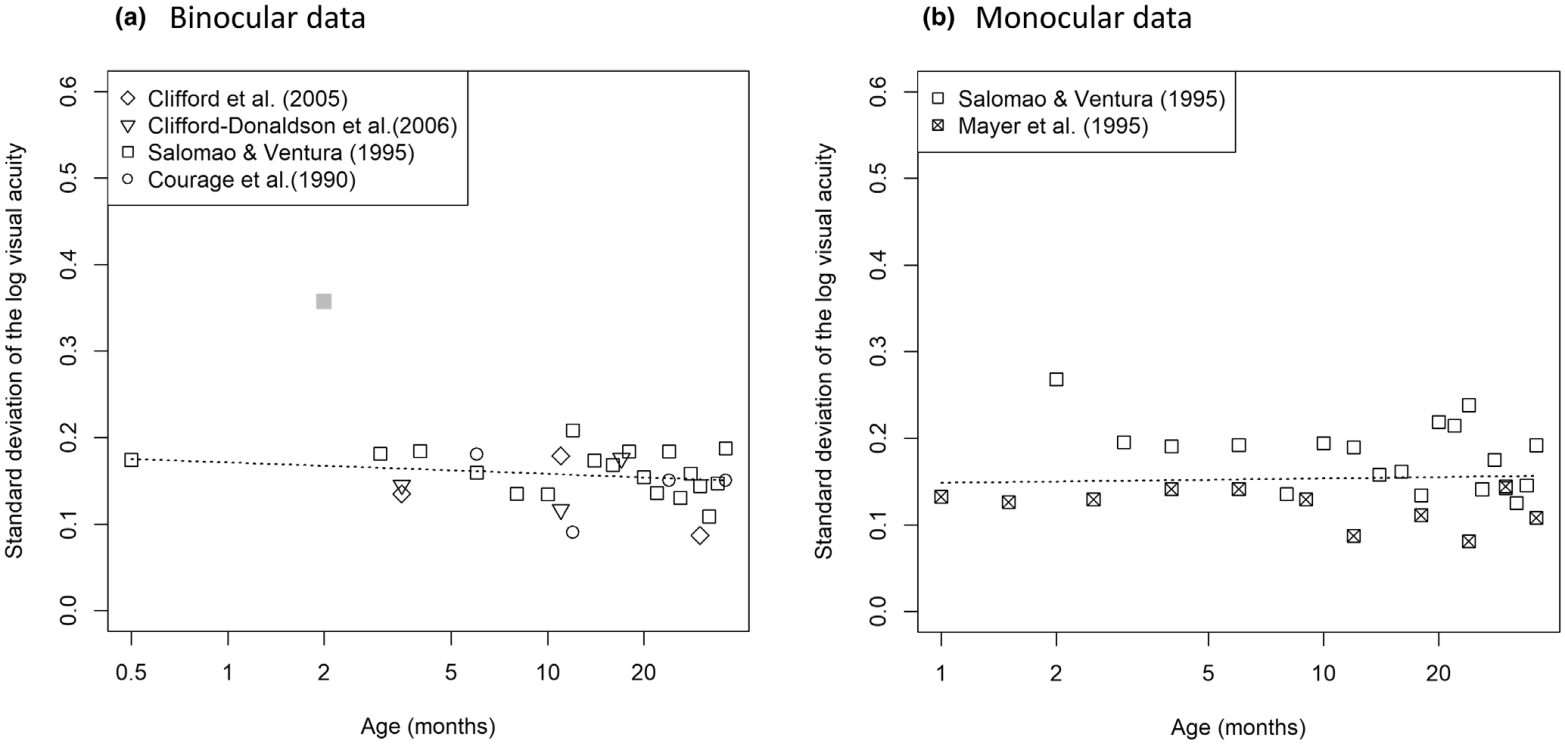
Standard deviation of the log visual acuity of the included papers plus a weighted linear regression fit. (a) Binocular data. (b) Monocular data.

Figure 1 shows the standard deviation of the log visual acuity as a function of age for binocular and monocular measurements, respectively. It is clear that the data point of Rios Salomao and Fix Ventura (1995) at 2 months of age is an outlier. This was also visible in the residuals and Q‐Q plot. We therefore excluded this data point from the analysis. The regression lines indicate that the standard deviation is quite constant as a function of age with a value of approximately 0.16. The slope of the regression lines was not statistically significantly different from zero (binocular: *p* = 0.37, *t* = −0.91; monocular: *p* = 0.78, *t* = 0.28).

**FIGURE 2:**
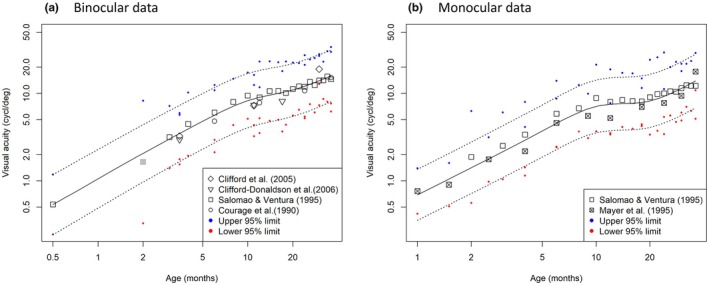
Binocular and monocular TAC II data from the included papers. If the papers had TAC I data, the data were corrected by 0.3 octave. Also shown is the fitted mean and 95% prediction interval based on weighted regression analyses. The binocular and monocular prediction intervals (reference limits) are given in more detail in Appendix A. (a) Binocular data. (b) Monocular data.

Figure 2 shows the binocular visual acuity data of the papers included in this study, the resulting mean regression line, and the upper and lower 95% prediction interval limits according to Equations 3 and 4. The values for both the lower and upper regression limits in Figure 2 are listed in Appendix A (Table A1), so it can be used to recreate these reference ranges. Furthermore, Appendix A also contains a figure that could be used for plotting data manually.

## 4 DISCUSSION

**FIGURE A1:**
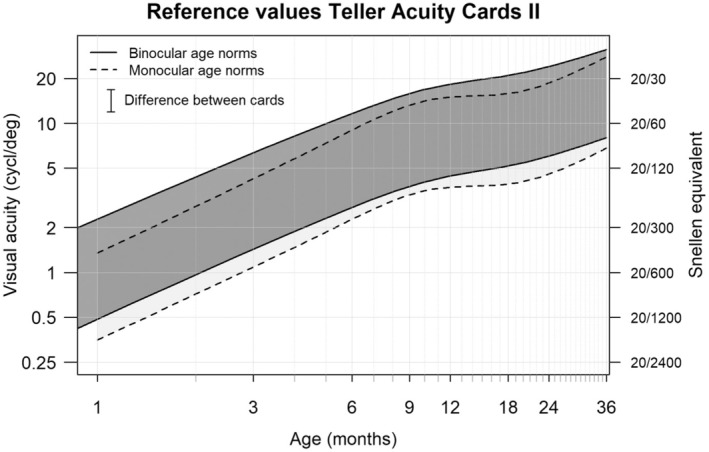
Reference ranges for the TAC II for both binocular and monocular viewing with a stage (little distraction). The ‘Difference between cards’ – bar shows the step size between consecutive cards of the test, which is half an octave (in other words, every second card the visual acuity doubles).

The assessment of visual acuity in infants and young children can present a challenge. An accurate visual acuity test is essential as a diagnostic tool, facilitating a rapid and precise diagnosis of abnormal visual development. Early detection of visual abnormalities is essential for the diagnosis of congenital and hereditary ocular disorders, to implement personalised care, and for facilitating the monitoring and optimisation of the child's development. A rapid diagnosis of inherited eye disease is important for prognosis, genetic counselling, and the possibility of therapeutic interventions. Our study focuses on the TAC test, the most widely used preferential looking method, and the gold standard for assessing visual acuity in children aged 0–36 months (Huurneman & Boonstra, 2016; Sarbajna et al., 2024). Visual development continues well into adolescence, with neural maturation further enhancing visual functions. But for children over 36 months of age, alternative assessments are more appropriate for evaluating visual acuity. The TAC II manual provides reference values, but these are not combined into one practical reference set and are based on the TAC I, the predecessor of the TAC II, which was discontinued in 2003 (Teller et al., 2003). Our meta‐analysis resulted in reference ranges for clinical practice (Figure A1) by combining earlier reference values with more recent studies and adjusting them to align as closely as possible with the TAC II charts. Consistent with the findings of previous studies (McDonald et al., 1986), our findings show a rapid growth phase in the approximately the first 8 months, followed by a less steep curve of visual acuity improvement. This phase of rapid growth in visual functions is linked to anatomical and physiological development of the eye and visual pathway, such as the increase in density of retinal cones or emmetropisation of the eye after birth (Leone et al., 2014; Yuodelis & Hendrickson, 1986).

Regarding the studies included in our meta‐analysis, we decided to exclude the study of Xiang et al. (2021) despite its compliance with the pre‐established criteria. Figure S1 illustrates the mean visual acuities from all papers that underwent full‐text review. The data from Xiang diverge significantly from both our findings and those of another study. Additionally, Xiang compares these results with existing norms established by Qiu (2011) , which we could not review due to its availability only in Chinese. The study of Xiang attributes the discrepancies in visual acuity to socio‐economic differences, a higher prevalence of refractive errors in children, and increased visual impairment in adults. However, we have reservations about whether these factors can account for such substantial differences in visual acuity in children aged 0–36 months. Another important note to make is that Clifford et al. (2005) and Clifford‐Donaldson et al. (2006) are papers originating from the same research group. After careful evaluation of the methods described in these papers, we concluded that there was no overlap in the study groups, and data came from separate trials.

A limitation of our study is that we did not identify a method to adjust for the use of a Teller‐acuity stage to reduce external distraction. Clifford‐Donaldson et al. (2006) compared the use of TAC with and without a stage, and only at the 17‐month measurement point did they find a significant difference (0.85 octaves lower visual acuity when not using a testing stage). At other ages, no significant difference was found. As this was the only paper on the use of a stage vs. not using a stage, we chose to only include studies with a stage. Nevertheless, we think that the reference set can be used by examiners who do not use a stage, providing they are aware that inattention due to interest in the surroundings might affect the outcome of the test, especially in toddlers. At this age, they may find the TAC charts to be less and less interesting. Leone et al. (2014) demonstrated that testability in children under 12 months of age was higher (≥85%) compared to children older than 12 months (≥ 65%) (Leone et al., 2014). When utilising the TAC, it is important to consider the child's stage of development. Clinicians should assess whether the TAC is still appropriate or whether an alternative visual acuity assessment using symbols, such as the LEA Charts, would be more suitable. This consideration is important, as older children may show a lack of interest in the TAC charts, potentially leading to inaccurate results that may underestimate visual acuity.

A notable strength of this study is the 0.3 octave adjustment made to align the original TAC data with the current clinical utilisation of the TAC II, thereby facilitating greater comparability. In recent years, advancements have been made towards the development of a computerised version of the TAC. This has involved the use of eyetrackers to measure gaze direction, thereby creating a more objective method of visual acuity that does not rely on the visual assessment of an observer. Wen (2022) tested the automated acuity card procedure and concluded the accuracy to be comparable to the TAC procedure. However, eyetrackers have certain limitations, and eyetracking can be challenging in children with nystagmus or iris transillumination (Wen et al., 2022).

In conclusion, this study provides clinicians with a unified set of reference values for the Teller Acuity Cards II. Accurate visual testing remains an essential element in diagnosing ocular disorders, and the Teller Acuity Cards II are the preferred method for evaluating visual acuity in children aged 0 to approximately 36 months. The integration of these standardised reference values into clinical practice will enhance diagnostic precision and support the early detection and management of visual abnormalities in this period of rapid visual development.

## CONFLICT OF INTEREST STATEMENT

The author reports no Conflict of interest.

## Supporting information


Figure S1.

## APPENDIX A Table and figure with reference limits for the tac ii test

**TABLE A1:** Data describing the visual acuity age norms of the TAC II as presented in Figure 2.

Monocular age norms			Binocular age norms		
Age (months)	Lower limit VA (cycl/deg)	Upper limit VA (cycl/deg)	Age (months)	Lower limit VA (cycl/deg)	Upper limit VA (cycl/deg)
1.000	0.353	1.351	0.500	0.241	1.173
1.169	0.414	1.587	0.602	0.291	1.403
1.366	0.485	1.864	0.725	0.352	1.677
1.596	0.568	2.191	0.873	0.424	2.004
1.865	0.666	2.576	1.052	0.511	2.392
2.179	0.781	3.032	1.267	0.616	2.853
2.547	0.917	3.572	1.526	0.741	3.400
2.976	1.078	4.212	1.838	0.890	4.045
3.478	1.269	4.973	2.213	1.067	4.805
4.064	1.496	5.881	2.665	1.277	5.697
4.750	1.767	6.967	3.210	1.526	6.740
5.550	2.090	8.268	3.866	1.818	7.956
6.486	2.473	9.812	4.656	2.161	9.368
7.580	2.891	11.506	5.607	2.562	10.999
8.858	3.292	13.144	6.753	3.023	12.853
10.351	3.600	14.416	8.133	3.518	14.819
12.096	3.749	15.061	9.796	4.000	16.687
14.135	3.795	15.293	11.797	4.403	18.195
16.519	3.839	15.516	14.208	4.735	19.379
19.304	3.988	16.170	17.112	5.072	20.562
22.558	4.347	17.679	20.608	5.519	22.164
26.362	4.937	20.143	24.820	6.156	24.486
30.806	5.765	23.592	29.892	6.986	27.525
36.000	6.824	28.015	36.000	7.998	31.210
