# Supplementary figures and images for: Reference values for the Teller Acuity Cards II (TAC II) in infants and preverbal children, a meta‐analysis
Source: Acta Ophthalmol. 2025 Jan 28;103(4):479–85. doi: 10.1111/aos.17447 (PMC12069961; doi:10.1111/aos.17447)

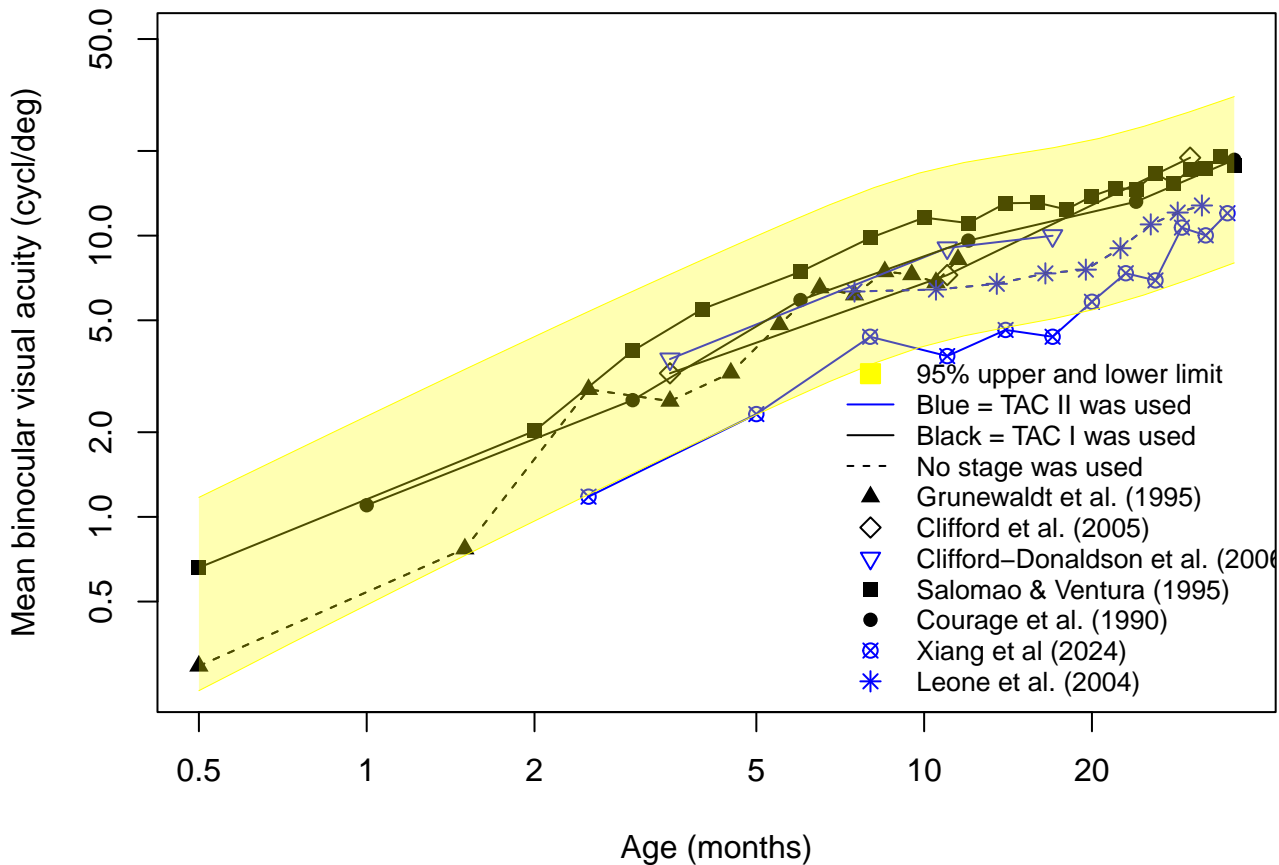

Supplement: Supplementary file 1 — Figure S1. [file AOS-103-479-s001.pdf]
